# Weight development from childhood to motherhood—embodied experiences in women with pre-pregnancy obesity: a qualitative study

**DOI:** 10.1186/s12978-024-01742-z

**Published:** 2024-01-30

**Authors:** Heidi L. Sandsæter, Trine Tetlie Eik-Nes, Linn Okkenhaug Getz, Hege S. Haugdahl, Elisabeth Balstad Magnussen, Janet W. Rich-Edwards, Julie Horn

**Affiliations:** 1 Department of Public Health and Nursing, Norwegian University of Science and Technology, Trondheim, Norway; 2 Department of Obstetrics and Gynecology, Levanger Hospital, Nord-Trøndelag Hospital Trust, Levanger, Norway; 3 Department of Neuromedicine and Movement Science, Norwegian University of Science and Technology, Trondheim, Norway; 4 Stjørdal Community Mental Health Centre, Levanger Hospital, Levanger, Norway; 5 Research Unit for General Practice, Department of Public Health and Nursing, Norwegian University of Science and Technology, Trondheim, Norway; 6 Levanger Hospital, Nord-Trøndelag Hospital Trust, Levanger, Norway; 7 Department of Obstetrics and Gynecology, St. Olav’s University Hospital, Trondheim, Norway; 8 Department of Clinical and Molecular Medicine, Norwegian University of Science and Technology, Trondheim, Norway; 9 Division of Women’s Health and Connors Center for Women’s Health and Gender Biology, Department of Medicine, Brigham and Women’s Hospital, Boston, MA USA; 10 Department of Epidemiologi, Harvard T.H. Chan School of Public Health, Boston, MA USA

**Keywords:** Adverse childhood experiences, Embodiment, Pre-pregnancy obesity, Weight history, Qualitative research

## Abstract

**Background:**

Pre-pregnancy obesity increases the risk of perinatal complications. Post-pregnancy is a time of preparation for the next pregnancy and lifestyle advice in antenatal care and postpartum follow-up is therefore recommended. However, behavioral changes are difficult to achieve, and a better understanding of pregnant women’s perspectives and experiences of pre-pregnancy weight development is crucial.

**Methods:**

We used a qualitative design and conducted semi-structured interviews with 14 women in Norway with pre-pregnancy obesity 3–12 months postpartum. Data were analyzed using thematic analysis.

**Results:**

Four themes addressing women’s experiences and understanding of their weight development were generated: (1) Unmet essential needs, (2) Genetic predisposition for obesity, challenging life course transitions and turning points, (3) Under a critical eye: an ever-present negative bodily awareness, and (4) Wrestling with food. Parents’ inability to meet children’s essential needs caused weight gain through an unbalanced diet, increased stress, and emotional eating patterns. Body criticism and a feeling of not belonging led to negative body awareness that influenced behavioral patterns and relationships. Participants reporting having had a good childhood more often described their weight development as a result of genetic predisposition, challenging life course transitions and turning points, such as illness and injuries. Nevertheless, these participants also described how eating patterns were influenced by stress and negative emotions.

**Conclusions:**

Healthcare providers should pay attention to the insider perspectives of pre-pregnancy weight development. An open and shared understanding of the root causes of these women’s weight development can form a basis for more successful lifestyle guidance.

**Supplementary Information:**

The online version contains supplementary material available at 10.1186/s12978-024-01742-z.

## Abstract (plain language summary)

Pregnant women with obesity face increased risks of pregnancy-related complications, warranting extended monitoring of their lifestyle and weight during pregnancy. The complexity of obesity makes lifestyle changes challenging both during and beyond pregnancy. Limited research exists on understanding weight development from the perspective of pregnant women with obesity. To explore their understanding and experiences of weight development from childhood to motherhood, we conducted in-depth interviews with 14 women with a BMI ≥ 30 before their pregnancies. The interviews were preformed 3–12 months post-birth. Through thematic analysis, four themes were developed: (1) Unmet essential needs, (2) Genetic predisposition for obesity, challenging life course transitions, and turning points, (3) Under a critical eye: an ever-present negative bodily awareness, and (4) Wrestling with food. Parental neglect of their children’s essential needs may result in unhealthy weight gain through an unbalanced diet and/or an urgent need to regulate negative emotions with food. Body criticism and self-perceived differences deprive children and adolescents of a carefree and accepting relationship with their bodies. While participants with a satisfactory childhood more often understood their weight in light of hereditary factors, difficult transitional phases, illness, or injuries, several of them described an eating pattern influenced by negative emotions such as stress, work pressure, and depressed mood. An open and shared understanding of the root causes of these women’s weight development can form a basis for more successful lifestyle guidance.

**Supplementary Information:**

The online version contains supplementary material available at 10.1186/s12978-024-01742-z.

## Background

Globally, the prevalence of overweight and obesity in children and adults has increased steadily in recent decades [1]. This also reflects weight development in reproductive age, where up to 50% of pregnant women are reported to have overweight or obesity in many low, middle, and high income countries [2]. In several European countries, the prevalence of pregnant women with obesity is close to 20% [3], making this one of the leading risk factors in obstetrics with adverse short- and long-term health consequences for mother and child [4–8]. This development increases pressure on the healthcare system and places a considerable personal burden on the individual [3].

Obesity development results from complex relationships between physical, biological, and psychosocial factors and their interplay [9, 10]. How these factors affect the development and maintenance of obesity in people is challenging to investigate due to the variation in factors that contribute to obesity within and between individuals [11].

Adverse childhood experiences (ACEs) have been consistently associated with overweight and obesity in children and adults [12–15]. Although bullying and peer rejection were not initially included in the original ACE study conducted by Felliti [12], later research exploring ACEs in relation to various health-related topics has incorporated these factors [16, 17]. Hemmingsson’s step-by-step model of obesity causation suggests that socioeconomic factors can increase psychological and emotional distress in childhood. Such emotional distress can in turn trigger a cascade of weight gain-inducing effects through allostatic overload and behavioral mechanisms aiming for stress regulation [18]. He suggests that acknowledgment of these root causes of weight gain could improve prevention and treatment programs [9].

Weight management interventions through lifestyle modifications continue to be disappointingly ineffective in both children and adults [19, 20]. One reason may be that problems with regulating emotions that drive relationships with food intake and choices can represent a significant barrier for successful lifestyle improvements [21]. Antenatal care is a preventive and health-promoting measure to provide pregnant women with lifestyle guidance, partly to prevent complications related to overweight and obesity and encourage positive lifestyle changes. In order for the guidance to motivate women, their emotional needs must be taken into account. However, several studies indicate that the topic of weight is often ignored or addressed in a way that women find stigmatizing [22]. This is often justified by time pressure, limited knowledge of healthcare personnel and barriers to talking about weight in pregnancy among both staff and pregnant women with obesity [23–25].

The antenatal and early postpartum period can be a “window of opportunity” where the pregnancy and transition to motherhood make women reflect on their childhood and their future wishes for their own health and that of their children [26, 27]. An open and unprejudiced approach to weight can be a step towards breaking the generational cycle, where children of parents with obesity are more likely to become overweight than children of normal-weight parents [28–31]. The approach and procedure of healthcare personnel during lifestyle assessments and guidance can benefit from knowledge from the “insider perspective” of women living with overweight and obesity. Understanding their views and feelings regarding factors that contribute to or protect against weight gain can lead to a more sensitive and open dialogue about weight during pregnancy and in the postpartum period.

There is still little research that explores people’s views and understandings of their weight history [32, 33], and we know of no studies among women of reproductive age. This study therefore aimed to enhance understanding of weight history from childhood to motherhood among women with pre-pregnancy obesity. We also wanted to explore whether and how these women understand and recognize ACEs (including bullying and peer rejection) as underlying factors in their weight development and how they understand and experience body and weight in the transition to parenthood.

## Method

We used a qualitative design and semi-structured interviews to elicit individual knowledge, understandings and experiences [34, 35]. The results were developed using a reflexive thematic analysis method, as outlined by Braun and Clark [36]. The research team consisted of a midwife (HLS), two obstetrician-gynecologists (JH and EBM), an intensive care unit nurse (HSH), a researcher specializing in obesity and eating disorders (TTEN), a physician and professor of behavioral sciences in medicine (LOG) and an epidemiologist (JWRE).

Stakeholders involved in the design of the research project included representatives of the Trondheim branches of the National Association for Overweight People, the Centre against Incest and Sexual Abuse Nord-Trøndelag and the Norwegian Women’s Public Health Association (Levanger youth branch), who provided input on the development of the interview guide and the information sheet (Additional file 2).

The study was approved by the Central Norway Regional Committee for Medical and Health Research Ethics (reference number:13.04.21/222481). All participants provided written informed consent.

### Sampling and recruitment

In Norway, free antenatal care is provided by midwives and general practitioners. Women with pre-pregnancy body mass index (BMI) ≥ 35 kg/m^2^ are offered referral to the nearest hospital maternity ward [37, 38]. Participants were recruited from women attending one of the three hospitals providing obstetric services in Trøndelag: St. Olavs Hospital (an urban university hospital), Levanger Hospital (a large local hospital) or Namsos Hospital (a small local hospital).

Potential participants were identified from the Hospital Trust’s journal system in Levanger, Namsos and St. Olavs Hospital using the International Classification of Diseases ICD-10 codes indicating antenatal care for pre-pregnancy obesity. Women were invited by a letter with an information sheet to participate in the interview 3–12 months after giving birth. Those who expressed interest in participation by SMS or email were contacted by telephone to arrange a date for an in-depth interview. To ensure attendance, a short SMS was sent to all participants the day before the interview.

The final sample consisted of 14 women who had given birth between December 2020 and April 2022 (11% of all invitees). All informants were aged 18 years or older, fluent in Norwegian or English and self-reported a pre-pregnancy BMI ≥ 30 kg/m^2^. The participants varied in age, parity, education, setting (rural, semi-urban or urban), marital status and birthing location. Twelve were born in Norway, one in another European country, and one outside Europe.

### Interviews

An open semi-structured interview guide was developed (see Additional file 2) and used during interviews. The interviews provided information for two studies. The data for the present study were drawn from the first part of the interview guide, which focused on women’s description and experiences of their childhood quality and their weight history until the time of the interview.

All interviews were conducted by the first author (HLS) from October 2021 to January 2023, at a location of the participant’s choice. Two took place in the participant’s home. One was a telephone interview, while eleven were face-to-face at the local university or in a hospital near the woman’s home. The interviews lasted for 70–120 min. During the interviews, the informants’ descriptions of their experiences were continuously validated against the interviewer’s understanding of their statements to ensure a well-rooted understanding of the data. Reflection on possible interpretations with the informant further improved common understanding of the data. Immediately after each interview, the first author wrote down her reflections to capture the overall impression, in addition to expected or thought-provoking phenomena. The interviews were audiotaped and anonymized while being transcribed verbatim.

After seven interviews, HLS conducted a preliminary analysis to identify patterns of meaning. This analysis was discussed with JH and HSH, who had previously read all interviews. The interview guide was updated to enable exploration of preliminary themes found during the first analysis. Seven more interviews were conducted before the dataset had the information power to answer our research topic [39, 40]. The women provided rich experiences and deep and varied descriptions. Most women gave multiple examples, making it easier to grasp the meaning of what they were describing.

### Data analysis

The analysis had an inductive, across-case approach, identifying themes from the data (a bottom-up orientation). Our preunderstandings of the research topic were rooted in Krieger’s, Merleau-Ponty’s and Leder’s phenomenological philosophy, understanding the body as “an integrated whole, embodied in the physical and biological surrounding world and in the sociocultural lifeworld” [41–43].

Due to the sensitive nature of the research topic and the potential difficulty for the women to express directly vulnerable aspects of their stories, we chose a flexible analysis method, reflexive thematic analysis, that is open for interpreting underlying meaning [40]. The analysis was led by HLS. All six phases in reflexive thematic analysis by Braun and Clarke: “familiarization, systematic data coding, generating themes, developing and reviewing themes and producing the report (results)” were discussed with the last author JH. The last three stages also involved TEN, LG and HSH, who had read a summary and reflection notes of each interview written by the first author (HLS). We have developed the themes we considered to represent the most valid story of the data. A codebook was used throughout the analysis to record all critical decisions and changes in this iterative process. Braun and Clarkes’ 15-point checklist was used to ensure the quality of the analysis [44].

## Results

The fourteen participants had varied background characteristics, see Table 1.

**Table 1:** Descriptive characteristics of the participants

Characteristics	n = 14
Age	
26–30	4
31–35	6
36–42	4
Marital status	
Married	5
Cohabiting	7
Single	2
Country of birth	
Norway	12
Abroad	2
Place of residence	
Urban	6
Semi-urban	3
Rural	5
Educational level	
Lower than high school	1
Certificate of apprenticeship	3
College or university	10
Occupational status	
Employed, full time	12
Employed, part time	1
Unemployed	1
Parity	
1	7
2	7
Pre-pregnancy body mass index	
Obesity class l (30- < 35 kg/m^2^)	4
Obesity class ll (35- < 40 kg/m^2^)	5
Obesity class lll (≥ 40 kg/m^2^)	5

They described a number of factors relevant to their weight history from childhood to motherhood. The participants’ experiences of how their lives had led to their obesity and the meaning of their life are expressed through the four themes developed in the analysis:

1. Unmet essential needs
2. Genetic predisposition for obesity, challenging life course transitions and turning points
3. Under a critical eye: an ever-present negative bodily awareness
4. Wrestling with food

Since obesity development is driven by several interacting factors, the themes are interwoven and not consistently mutual exclusive. An overview of codes, subthemes and themes is presented in Additional file 1: Table S1, Additional Material.

### Unmet essential needs

Unmet essential needs was a consistent theme. These could be physical needs such as access to “proper” food and adequate physical activity. There were also unmet emotional and social needs, such as a lack of secure adult relationships and good friends. Several participants felt that these needs were unmet throughout most of their childhood.

Growing up with unhealthy eating habits and little physical activity was described in connection with early weight problems in childhood. Parental lack of interest and knowledge about food, the mother’s diet, poor finances and little time were all described as having influenced the family lifestyle: “…*My dad never thought about the food he served up. Mom was never a gourmet cook either. With five children and little money, it was just as simple as possible as long as it was dinner. It was just quick and easy*” **Informant 1.** *“I’ve realized that I probably grew up on pasta and hot dogs*” **Informant 4.**

Loyalty conflicts were described in connection with parental divorce conflicts. Consequently, some felt a loss of support and close contact, especially with their father. Despite regular contact, they felt replaced and pushed to the sidelines. Several had grown up with insufficient close contact and care from one or both parents. Repeated criticism gave several participants a feeling of being worthless: *“What you did was never good enough and they laughed at you if you made a mistake… I ended up behind the others at school, socially and in everything. Bullying, parents arguing, divorce kid, everything*” **Informant 1.**

A high level of conflict in the family led to insecurity and stress. One participant was often afraid that her father would beat her younger siblings because they did not follow the countless rules at home. She felt responsible for preventing her father’s angry outbursts, e.g., by keeping order in the house until he came home.

“You never knew what mood he’d be in when he got home. So we were forever walking around and sensing his mood. Is it safe to act like a child now or should we keep away? Yes, we were afraid when mom was at work or out shopping, because he was unpredictable and unsafe, and often angry about things that we couldn’t predict he’d be angry about.” **Informant 3.**

Several informants described mental health problems in childhood and the feeling that their parents did not see or could not meet their needs for additional security and support. Insecure relationships with parents were an obstacle to expressing anxiety and depression, in addition to fear of not living up to the parents’ expectations. One participant did not seek support from her mother for her insomnia and restlessness because she knew how her mother would gloss over her problems. The participants now understood that the anger they felt as children was due to all the worry and anxiety they often had to deal with alone.

To keep up their facade, some participants’ families refused to seek help for children’s mental health or weight problems. One woman, for example, was not diagnosed with attention deficit disorder until adulthood because her father refused to have her assessed. It was difficult to realize that the shame their parents felt was due to their mental health or weight problems. One woman whose weight greatly increased due to severe depression said:

“My mum wasn’t afraid to tell me she was ashamed to go out with me if we met people she knew. She was ashamed to walk with me, and she focused on what other people thought: ‘I’m sure no one recognizes you.’ Lots of nasty comments like that.” **Informant 8.**

Lack of close friends, harassment and unwanted attention to their body and appearance had made some of the informants feel lonely, insecure and often vulnerable as children. They described how they needed to “just keep going” at school and in leisure time. Unmet essential needs in childhood increased the urge to regulate difficult emotions with food. These stresses were present throughout most of their childhood, with no access to resources that could ease their burden. They were at the mercy of negative family relationships and/or a destructive school and leisure environment. One woman had had meetings with the school nurse throughout her schooling, but her home situation and the bullying were never addressed. Even long-term support from a professional did not enable her to escape from a destructive environment. To get away, some moved in with boyfriends or other family members in the vulnerable teenage phase:

“I did what I wanted, I couldn’t bear to listen to them [her parents], I felt like we had no relationship. They went off on vacation by themselves. I started moving out when I was 15 or 16. I didn’t want to live at home. Since then I’ve had older boyfriends I’ve lived with. That certainly hasn’t made me careful about alcohol or food.” **Informant 4.**

### Genetic predisposition for obesity, challenging life course transitions and turning points

This theme displays other factors participants highlighted in their weight development. Challenging life transitions encompass physiological and emotional changes during puberty and other life phases. "Turning points" refers to major events like personal or loved ones' illnesses, injuries, or deaths. Women who grew up without emotional and physical neglect realized more clearly that genetic predisposition for obesity, challenging life course transitions and turning points were important factors in their weight history. Despite a normal diet and activity level, they put on weight faster than their peers. They understood hereditary predisposition as biological factors such as body type, slow metabolism, polycystic ovary syndrome, early puberty, psychological problems or a good appetite. Many participants also saw similarities in body shape between themselves and other overweight family members. An understanding of obesity as inherited means that weight development is viewed as predetermined, difficult to change and not self-inflicted: “*I have bad genes when it comes to weight. I have practically no metabolism…[….]… We have a lot of problems with metabolism in my family. And it goes way back. Several of us have inherited a goiter, going a long way back”* **Informant 5.**

The participants emphasized puberty and youth as a challenging transitional phase of their weight history. Many self-identified as big but within the normal range until puberty and traced the start of their weight problems to puberty. Body changes such as growth and physical maturation were consistently viewed as negative. Weight changes were sometimes attributed to biology, or to altered habits in physical activity, food and alcohol. For some, physical development led to unwanted negative attention from boys. “*I was the first one to get a grown-up body. I was in sixth grade, I think. Then I got so… bullied. …for me it felt like they thought I could handle so much more physically. So there was a lot of hitting and kicking and pushing by the boys”* **Informant 9.**

Other difficult transitional phases were starting college and moving out alone or with a boyfriend. Many of the women could not maintain healthy routines at this time, due to for example, studying hard.

Injuries and illnesses were turning points in some informants’ weight trajectories, with periodic restrictions on physical activity. Some had to give up their favorite sport or switch to other forms of exercise. Injuries often took place in adolescence or young adulthood, when some found that inactivity made it more difficult to maintain healthy food habits. Several of the women saw these events as clear milestones when their weight escalated. One of them reported a huge weight increase in association with chronic fatigue syndrome following an episode of virus-induced vertigo in her late teens: “*After I got sick, I put on 20 kilos. And it didn’t take long. From being active and going to work, and working a lot and exercising a bit, to being completely bedridden, to feeling so nauseous that the only thing you can eat is potato chips and stuff like that*” **Informant 11.**

Disease periods had a major impact on the weight development of several participants. The direct effects of ill health and medication affecting their appetite and metabolism, such as steroids or psychotropics, led to a clear weight gain at the time of the illness. Some went from normal weight to severe obesity in a few years. A woman who suffered from anxiety in childhood became severely depressed after high school in connection with a stressful event in her family. Psychotropic drugs increased her appetite and radically changed her food preferences. She changed from underweight to severely obese in her early twenties. She explained the side effects of psychotropics as follows:

“It’s like going around being hungry all the time. Only that in the evening when you’ve taken the medicine it’s … well, it’s not just food you crave. It’s bad food, it’s sugar and when you start eating it, it tastes completely different. It tastes much better. Normally, you might feel nauseous and vomit from the amount and type of food. But you lose that ability… there’s no end to it.” **Informant 8.**

Illness in the family, loss of close family members or stressful care situations were also events that disrupted good routines and increased the need for comfort eating. One woman said that food provided reassurance when she lost several family members during the COVID-19 pandemic.

### Under a critical eye: an ever-present negative bodily awareness

Some participants had experienced direct or indirect body criticism since childhood. Weight, body and appearance were a constant source of criticism, exclusion and bullying at home and at school. Insinuations by parents that they could eat different foods or exercise more were interpreted as an encouragement to diet. As a result, some participants experienced a strong sense of shame and loathing of their body. Their own desire to lose weight was described as a sign of poor self-acceptance, which also caused shame, and made them feel more vulnerable in relationships with others. Imagining what others thought about their weight increased their body consciousness: “*I might meet people, and then I know what they’re thinking. Okay, I’m ugly, disgusting, you’re like this and like that and… But I don’t think anyone can think such nasty thoughts about me as I do myself”* **Informant 3.**

Body criticism and weight bullying meant that negative body awareness was all-pervasive from childhood. The informants’ descriptions show how they had to deal with their bodies at an early stage. Bodily awareness lost its natural place in the background of consciousness and entered the foreground. Many of life’s natural activities were reduced to dealing with the body and its appearance. The informants could not forget their bodies through play and spontaneous joy. Their self-consciousness constrained them and the critical gaze of others prevented them from showing their potential. Meals were no longer just a matter of eating because they were hungry. They learned to hold back. Food was reduced to a question of body. Their self-consciousness pushed them out of their comfort zone and into an attitude of self-assessment, insecurity and stress: “*I can’t ever remember feeling comfortable since I realized I had a body. I’ve never been comfortable in that body because it’s been different from my friends’ bodies. Before I started thinking about my body, everything was fine*” **Informant 3.**

Informants also discovered how different they were when they compared their size with that of their peers, due to the difficulty of finding fashionable clothes in large sizes, or when they saw pictures of themselves. “*We were on holiday that summer. Mom and Dad took pictures, and when we developed the ones from the water slide, I saw my stomach. And that was it. Then something in my head said now you’re not like everyone else. Now you’re, well… now you’re fat*” **Informant 9.**

Some informants found that boys preferred slim girls, which also increased their body awareness and feeling of inadequacy. One said that she was invisible to boys in her youth. In her twenties, she lost weight for a period and had the experience of being attractive. This, however, led to destructive behavior where she sought acceptance in night clubs at weekends. Her desire for acceptance meant that on some occasions she was exposed to what she later would call abuse:

“When I lost weight and I was alone again [after breaking up with her boyfriend], I kind of went back a bit to ‘feeling invisible’ [from the time before she got a boyfriend]. So then I went to town and I met men who were after one thing. And then they got that one thing. Then I carried on like that for quite some time until I realized it wasn’t healthy. It was actually a kind of self-harm (….) Difficult situations that would definitely qualify as rape these days.” [Referring to that period] “I actually think it’s a bit strange, or in fact it’s not strange. I felt fine, in fact. I wanted confirmation that I was now finally attractive enough.” **Informant 9.**

Several of the women saw their weight as a sign that they had failed in an important area of life. Some compensated by being more successful in other areas, such as education and work. However, although a good education or a good job compensated for their feeling of failure in losing weight, some described how a long education or a demanding job were very sedentary. Being highly extroverted to fit in with family and friends was in retrospect seen as an unconscious “obesity compensation strategy” that forced them into an artificial role, robbing them of their natural self.

“I kind of wanted to make up for not having much success in losing weight. I wanted to get good grades at college instead. Perform better. But then I sat in the reading room all day. I wanted to perform in other areas, to catch up, quite simply. But that didn’t really help things either.” **Informant 12.**

Body criticism or feeling different was described as a constraining form of self-consciousness by some participants, and those without such negative experiences tended to feel better about their bodies. Their body more often seemed to be in the background, and they did not let themselves be defined in terms of weight. They described themselves as strong and robust, and had found that they were just as fit and able to work as many women of normal weight. Many had had normal pregnancies and births without requesting sick leave. They saw this as proof that they were in good health and that their weight had not prevented them from living a normal, active life: “*Throughout my whole pregnancy, I didn’t have any kind of elevated blood pressure, or any complications or any getting sick. I was fit and fine until the last day.*” **Informant 10.**

An obese woman’s attitude towards her body can improve during pregnancy. When the pregnancy became visible, several participants said they felt fine. One woman who usually hid her body from view said that she did not mind it so much if other people looked at her when she was pregnant. “*I felt that for once I didn’t think it was so awful that people looked at my body. Because then my tummy wasn’t big just because I’m fat, it was big because there was a baby inside.*” **Informant 7**. In that way, pregnancy and motherhood can make some women escape from negative body consciousness. Body awareness is shifted towards the baby, and they now have their own family where they belong. Nevertheless, some women could still feel a deep aversion to their parents’ critical gaze.

### Wrestling with food

Informants reported how food could meet various needs, and terms such as comfort eating, snacking and overeating were used. Several women described eating patterns resembling binge eating with loss of control, secretly eating large amounts of food rapidly until they felt nauseous. These binge episodes were followed by shame and distress. Family meals during childhood were described as difficult and even toxic by some. Hiding what they ate to avoid criticism at mealtimes often started in early childhood. There was an atmosphere of fear where the women described a fear of “eating the wrong food”. Some described ambivalent feelings towards food. On the one hand, food was something forbidden which filled many with regret and fear of weight gain, ill health and death. On the other hand, food was enjoyable, alleviated their difficult feelings, gave them a respite and helped to fill their inner void. Eating could be seen as a repetitive cycle triggered by difficult emotions. Eating provided brief moments of pleasure, before they were again overwhelmed by negative emotions:

“I don’t think anyone really knows. As an adult, I have thought maybe it’s a kind of bulimia without the vomiting. You do it in secret. I expect they’ve made some diagnostic codes for this overeating… I’ve lived alone for a very long time. So there was nobody to watch what I ate, so I could eat whatever I wanted. And then you don’t feel ashamed either… But I don’t dare to eat a lot in front of my mother. Getting a comment or a look, or… You infer all kinds of things from what people say, which might not even be there.” **Informant 4.**

Some of the interviewed women described their childhood as safe and predictable while revealing how, later as adults, they had come to focus their lives around food to deal with stress, depression, loss and other problems. Eating compensated for something missing in periods of their lives, such as when they felt lonely, slept badly or lacked energy. Conversely, food could also compensate when there was too much of something, such as stress, caring responsibilities and work pressure. Many women were afraid that starting full-time work after maternity leave would trigger poor eating habits.

One of the participants who grew up in a non-Western culture described how her weight shot up in Norway after she lost close family members to illness “*I could find comfort in food (…) I ate my feeling away (…)*” **Informant 10.** She felt that it would have been impossible for her to use food to comfort herself in her home country, because of the food culture and way of life that require time and preparation, unlike in Norway where she felt that ready-made food means that meals will always be ready in five minutes.

Extensive body criticism and excessive focus on food and diets initiated by their mothers led to further weight gain when women moved away from home. Eating without the critical gaze of their family was a relief, and enabled them to eat what they wanted. However, others found that the loss of parental support and routines paved the way for eating disordered behaviors that they had not had previously.

Many of the women wanted to prevent their own past from becoming their children’s future. They focused on enabling their child to have a healthy lifestyle and a normal relationship with food and body:

“When you have a child, you have responsibility. On my husband’s side of the family there’s a lot of obesity. If we mix that with a mother who doesn’t do her job properly, then I’m afraid my daughter might become overweight, and I don’t want that… I don’t want her to feel heavy and fat, so she can’t join in things. I don’t want her to feel pain and get sick. That’s what’s important to me. So I’m sure I will watch out for that.” **Informant 13.**

By providing emotional support and being reliable and predictable mothers, the women wanted to help their children develop a positive self-image, body satisfaction and a sound relationship to them as parents. These factors were lacking for several women in this study, and they now realize in retrospect how important they were for their weight development.

## Discussion

The results of this study of women with pre-pregnancy obesity offer an insider perspective on their weight history from childhood to motherhood. To summarize, it seems that neglect of physical, social, and emotional needs results in unhealthy weight gain through an unbalanced diet and/or an urgent need to regulate negative emotions by eating. Body criticism and self-perceived difference deprive children and adolescents of a carefree and accepting relationship with their body. Negative body awareness affects their actions and relationships later in life. Although participants with a satisfactory childhood more often understood their weight in light of hereditary factors, difficult transitional phases and illness or injuries, several of them described an eating pattern influenced by negative emotions such as stress, work pressure and depressed mood.

Despite the insights of the researchers who developed the ACE study [12–15], the medical profession has long neglected to deal with the complex, biopsychosocial causes of obesity. Only in recent years has a more nuanced overall picture begun to emerge [9, 45]. In order to further develop this picture, valid theoretical concepts are needed. Social epidemiologist Krieger has helped to refine ways of understanding and applying the concept of embodiment in the generation of new knowledge, illustrated with this statement: “Embodiment, as a multilevel phenomenon, integrating soma, psyche, and society, within historical and ecological context, and hence an antonym to disembodied genes, minds, and behaviours” [41].

With his phenomenological philosophy of the body, Merleau-Ponty represents a break with the biomedical dichotomization of the relationship between body and soul, psyche and soma. He describes how we experience the world with our bodies, both physically and emotionally [46, 47], and these experiences have been found to involve the same biological systems in the body [18]. Merleau-Ponty and Leder both describe how the body alternately appears in the foreground and the background of our consciousness, depending on whether it is perceived to be functioning or affected by illness or injury [42, 43]. In line with this, our study shows that body criticism, bullying and exclusion, and the consequent bodily self-criticism, shift the body from the background to the foreground of a person’s consciousness, just as in illness and injury, leading to a new body awareness [42, 47–49]. This body awareness was described by some participants as inhibiting their emotional health and quality of life.

Our results are supported by some quantitative studies reporting that women who experienced negative weight-related comments from parents or peers were more likely to develop emotional eating patterns to deal with negative feelings. Comments on body and food affect young people’s self-esteem and feeling of belonging, which again impacts their emotional health with increased depression, anxiety and symptoms of binge eating disorder [50–52]. Further, emotional abuse has demonstrated moderate to strong specificity in predicting binge eating [53]. A qualitative study of Norwegians with severe obesity found that the participants felt that their large body size was “shaped by their childhood”. Participants described how food compensated for the lack of an intimate relationship [54], which supports our findings that insecure relationships lead to emotional hunger, involving food as compensation. Conversely, it has been shown that positive childhood experiences, including close friendships with peers, can increase resistance to obesity development in children and adolescents with ACEs [55, 56]. Several women in our study lacked these protective factors and described a combination of an emotionally insecure home environment and a lack of close friendships in childhood. In our study, women without these negative experiences expressed greater satisfaction with themselves, more often seeing their weight problems as due to genetics, difficult life course transitions and intrusive events.

A qualitative study from Denmark by Smith et al. explored subjective understandings of life-course weight trajectories among middle-aged Danish men and women [32]. For the female participants, transitional phases linked to women’s biological life cycle, such as puberty, were seen as vulnerable phases when the women put on considerable weight. Women in our study also mentioned puberty as a weight transition. The Danish study also found that disruptions in close social relationships with parents, friends or boyfriends led to weight gain by altering the person’s emotional support and negatively affecting her mental health [32]. These findings concur with our findings that insecure relationships and poor emotional support could all lead to undesired weight gain. Explanations for weight gain such as those described by Smith et al. as typical of men correspond with the experiences of many of the women in our study. This applies to weight gain in connection with shifting commitments in education, work, and family life and to periods of injury, resulting in reduced levels of physical activity. Many of the participants in our study were well educated and had high work demands. The results that Smith et al. described as gender-specific can also be seen as a result of heavy workload and family obligations.

Further, side effects of drugs such as psychotropics were highlighted by the women in the Danish study as significant turning points in their weight history [32]. Drug-induced obesity is a well-known phenomenon primarily related to the use of psychopharmaceuticals or steroids, which also affected several of the women in our study [10, 57].

Unlike our findings, the study by Smith et al. emphasized that female participants with different levels of education described their experiences differently. Women with a high educational level more often described their weight problems within a depth-psychological framework, where e.g., poor self-image was seen as a result of a lack of love and adequate care in childhood [32]. In our study, several women explained their weight gain in this way, irrespective of educational level.

In our study, important transitions in life such as moving in with a partner were emphasized as periods when the women gained weight, referred to by several with a Norwegian expression meaning “partner-induced kilos”. This is supported by a study that found that women, but not men, acquire more unhealthy eating habits when they begin to cohabit [58].

Most of the participants in our study felt that genetic factors had influenced their weight development. Hereditary predisposition was seen as a contributing factor in a complex picture, representing a vulnerability where many participants felt that they shared genes for rapid weight gain with several members of their close family. This understanding is supported by studies and literature that have examined associations between genetic predisposition and the development of obesity [10, 59], where the intergenerational transmission of obesity is particularly well documented [29, 60].

Hemmingsson developed the understanding of intergenerational transmission and pointed out the importance of children’s home environment. He described how low socio-economic status increases the level of conflict in families, which leads to neglect of children’s social and emotional needs. This can result in chronic stress that affects children’s energy balance and leads to weight gain [9]. This explanation concurs with our findings, where several women described how insecure relationships and unmet emotional needs led to a disordered eating pattern that then resulted in weight gain. Several participants were subject to stigma and bullying because of their weight, which increased their need to eat to compensate for their distress. Our study included participants with high and low socioeconomic status, but due to the qualitative design, we cannot draw any conclusions about differences in childhood environment or life experiences between the two groups.

A number of women in our study found that body pressure decreased during and after pregnancy, and several felt more satisfied with their bodies when their pregnancy was visible. This was due to greater tolerance by others of a pregnant body that deviated from the ideal body. This is supported by findings from a meta-analysis suggesting that some women with obesity find that it is socially acceptable for a woman to have a larger body size when pregnant [61]. A longitudinal mixed methods study compared changes in body image throughout pregnancy. Positive changes were mainly experienced by women with overweight or obesity. Feeling less self-conscious and more positive about public scrutiny was a common explanation. Many women with overweight or obesity felt that their bodies had become more physically attractive through pregnancy, which in turn increased their feelings of confidence [62].

## Strengths and limitations

Our results provide an overall picture of how women in Trøndelag with pre-pregnancy BMI ≥ 30 understand their weight history in a retrospective perspective.

Our sample was strategic, with broad variation in important baseline characteristics such as age, marital status, parity, education level and different obesity classes. Although most women recruited were highly educated, their experiences did not seem to differ from those of women with a lower educational level. This overrepresentation may suggest that highly educated women are more inclined to engage in self-reflection regarding their weight history. We were only able to recruit one woman who was born and raised outside Europe, which is a limitation of our study.

All interviews were conducted within one year postpartum, when the women still had their pregnancy fresh in mind, while providing a perspective from early parenthood. The study was conducted by a multidisciplinary research team, which has enabled a view of the thematic analysis from several perspectives, leading to discussion of alternative understandings of the results.

## Conclusion

The insider perspective of personal obesity development from childhood to motherhood shows what women who have recently given birth consider to be important factors in their pre-pregnancy weight history. The findings can encourage healthcare services in general and maternity care in particular to better understand pregnant women with obesity and adopt a patient-centered focus in providing care to them. A perspective that many women recognize, with a stronger focus on the root causes of their obesity rather than merely focusing on the present situation, can provide a better basis for lifestyle counseling. Healthcare personnel in pregnancy and maternity care may also benefit from knowing that for some obese women, pregnancy may be the first time in their lives that they are satisfied with their bodies, after years of negative body awareness. It is important to recognize and acknowledge this change in order to avoid a new humiliating ‘objectification’ of the pregnant woman through a standardized, insensitive weight management approach in the consultation. Lifestyle talks should not be ignored for fear of offending pregnant women with obesity, but women should be invited to an open dialogue free from prejudice (Additional file 3).

Further research might explore how a change towards more positive body awareness can be utilized in health promotion aimed at women with a high pre-pregnancy BMI.

### Supplementary Information

**Additional file 1: Table S1.** Codes, subthemes and themes

**Additional file 2: **Interview guide.

**Additional file 3: **Questionnaire.

## Data Availability

The datasets analyzed during the current study are not publicly available because the participants have not consented to sharing the interview material.

## Abbreviations

ACE: Adverse childhood experience
BMI: Body mass index
