# Supplementary material for: Weight development from childhood to motherhood—embodied experiences in women with pre-pregnancy obesity: a qualitative study
Source: Reprod Health. 2024 Jan 30;21:15. doi: 10.1186/s12978-024-01742-z (PMC10826130; doi:10.1186/s12978-024-01742-z)
Supplement: Supplementary file 1 — Additional file 1: Table S1. Codes, subthemes and themes [file 12978_2024_1742_MOESM1_ESM.pdf]

Table S1. Codes, subthemes and themes

Codes	Subthemes	Themes
-Divided loyalties in parental divorce conflicts<br>-Impossible to satisfy both parents<br>-Replaced and pushed to the sidelines by the father's new family<br>-Keeping up the family facade, leading to shame<br>-High level of conflict<br>-Unpredictable parents<br>-Direct or indirect body criticism by parents<br>-Unpredictable relationships with friends<br>-Peer harassment<br>-Poor family eating habits<br>-No help for unhealthy eating habits and lifestyle	-Insecure relationships<br>-Unmet emotional and social needs<br>-Fear of not being good enough<br>-Unmet physical needs for healthy food and adequate exercise<br>- To be in a state of preparedness/ Between a rock and a hard place	Unmet essential needs
-Bad genes<br>-Puberty as a vulnerable phase: changes in habits and biology<br>-Moving away from home<br>-Living with a partner<br>-Education<br>-Illness and injuries (self or close family member)<br>-Effect of medication on weight	-Hard to fight a genetic predisposition for weight gain<br>-Vulnerable transitions<br>-Life-changing experiences<br>-Altered life situation disturbed routines<br>-Injuries and illnesses as turning points	Genetic predisposition, challenging life course transitions and turning points
-Toxic body criticism<br>-Being put on a diet<br>-Can't find clothes in your size<br>-The photo proves it: now you're fat<br>-Can't join in play like the others<br>-You become awkward and stiff<br>-To put on a happy mask<br>-Strong and robust<br>-Weight is no obstacle<br>-Being seen as a whole person	-Body consciousness and realization<br>-Being defined by your weight<br>-Self-objectification and objectification by others<br>-Obesity compensation: succeeding in other areas<br>-Feeling unfree and forced into a role<br>-Being prevented from showing your potential<br>-Feeling good in your body, your weight doesn't define you as a person	Under a critical eye: an ever-present bodily awareness
-Emotional eating patterns<br>-Loss of control over eating<br>-Food as a respite<br>-Food as compensation	-Emotional hunger	Wrestling with food
