# Supplementary material for: Weight development from childhood to motherhood—embodied experiences in women with pre-pregnancy obesity: a qualitative study
Source: Reprod Health. 2024 Jan 30;21:15. doi: 10.1186/s12978-024-01742-z (PMC10826130; doi:10.1186/s12978-024-01742-z)
Supplement: Supplementary file 2 — Additional file 2: Interview guide. [file 12978_2024_1742_MOESM2_ESM.pdf]

Interview guide S2
Weight development from childhood to motherhood: Embodied experiences in women with pre-pregnancy obesity- a qualitative study

Introduction to the interview:

Information on confidentiality applicable to interviews. All data will be anonymized and stored according to approved guidelines.

You can decide yourself how much information you want to share. You can interrupt the interview at any time for a break or to terminate it. (Agree on signs for the person to give when she needs a break or wants to end the interview).

I may take notes during the interview or take short breaks to find natural follow-up questions. The interview may take about 60-90 minutes. No answers or stories are right or wrong; what is important is that you talk about your own personal experiences. Please give examples.

1) Please tell me a little about yourself
2) Childhood and adolescence
3) Thinking back to your childhood/adolescence, how would you describe it?

   - How would you describe your family situation during your childhood?
   - Who was there for you and in what way?
   - Many women have adverse experiences in childhood, adolescence and later life. How has it been for you? (emotional, physical or sexual experiences)

4) Please describe your weight history from as far back as you can remember.
5) Food: For many people, food is more than just food, how is it for you?
6) Body awareness and self-image
7) We have carried out a study showing that difficult experiences in childhood affect women's weight. How has that been for you?

Topics that stood out in several interviews and in the first preliminary analysis. These topics should be explored in all new interviews.

1) Body criticism and harassment
2) Lifestyle in childhood and today
3) The importance and function of food in childhood and later
4) Vulnerability, resilience, vulnerable periods for weight gain
5) Self-image and self-confidence, functioning and health in the future
6) Responsibility and guilt
