# Supplementary material for: Weight development from childhood to motherhood—embodied experiences in women with pre-pregnancy obesity: a qualitative study
Source: Reprod Health. 2024 Jan 30;21:15. doi: 10.1186/s12978-024-01742-z (PMC10826130; doi:10.1186/s12978-024-01742-z)
Supplement: Supplementary file 3 — Additional file 3: Questionnaire. [file 12978_2024_1742_MOESM3_ESM.pdf]

Questionnaire S3. Weight development from childhood to motherhood: Embodied experiences in women with pre-pregnancy obesity- a qualitative study

Date:

First name:

Age:

Place of residence: Rural ☐ Semi-urban ☐ Urban ☐

Marital status: Married ☐ Cohabiting ☐ Single ☐

Highest completed level of education: Primary/lower secondary ☐ Upper secondary ☐ Vocational qualification ☐ Higher education ☐

Work/job:

Full- or part-time (when not on maternity leave):

Full-time ☐ Part-time ☐ Other ☐

Weight at start of pregnancy:

Who gave you pregnancy check-ups? Midwife ☐ Your doctor ☐ Combination ☐

Height:

Number of children:

Delivery method: Normal delivery ☐ Vacuum/forceps ☐ Planned cesarean ☐ Unplanned cesarean ☐

Emergency cesarean ☐

How many weeks pregnant were you at delivery?

Feeding: Breast-feeding: Yes ☐ No ☐ Number of months of breast-feeding Bottle-feeding: Yes ☐ No ☐

Combination of breast- and bottle-feeding ☐

Please add any comments here:
